# Nitrate decreases ruminal methane production with slight changes to ruminal methanogen composition of nitrate-adapted steers

**DOI:** 10.1186/s12866-018-1164-1

**Published:** 2018-03-20

**Authors:** Liping Zhao, Qingxiang Meng, Yan Li, Hao Wu, Yunlong Huo, Xinzhuang Zhang, Zhenming Zhou

**Affiliations:** State Key Laboratory of Animal Nutrition, College of Animal Science and Technology, China Agricultural University, Beijing, 100193 People’s Republic of China

**Keywords:** Nitrate, Methane, Methanogen diversity, Hiseq sequencing

## Abstract

**Background:**

This study was conducted to examine effects of nitrate on ruminal methane production, methanogen abundance, and composition. Six rumen-fistulated Limousin×Jinnan steers were fed diets supplemented with either 0% (0NR), 1% (1NR), or 2% (2NR) nitrate (dry matter basis) regimens in succession. Rumen fluid was taken after two-week adaptation for evaluation of in vitro methane production, methanogen abundance, and composition measurements.

**Results:**

Results showed that nitrate significantly decreased in vitro ruminal methane production at 6 h, 12 h, and 24 h (*P* < 0.01; P < 0.01; *P* = 0.01). The 1NR and 2NR regimens numerically reduced the methanogen population by 4.47% and 25.82% respectively. However, there was no significant difference observed between treatments. The alpha and beta diversity of the methanogen community was not significantly changed by nitrate either. However, the relative abundance of the methanogen genera was greatly changed. *Methanosphaera* (P_L_ = 0.0033) and *Methanimicrococcus* (P_L_ = 0.0113) abundance increased linearly commensurate with increasing nitration levels, while *Methanoplanus* abundance was significantly decreased (P_L_ = 0.0013). The population of *Methanoculleus,* the least frequently identified genus in this study, exhibited quadratic growth from 0% to 2% when nitrate was added (P_Q_ = 0.0140).

**Conclusions:**

Correlation analysis found that methane reduction was significantly related to *Methanobrevibacter* and *Methanoplanus* abundance, and negatively correlated with *Methanosphaera* and *Methanimicrococcus* abundance.

**Electronic supplementary material:**

The online version of this article (10.1186/s12866-018-1164-1) contains supplementary material, which is available to authorized users.

## Background

Globally, around 40% of anthropogenic methane from the agricultural sector is produced by livestock [1] and methane production results in approximately a 2-5% energy loss from feed [2]. Several additives have been tested to lower methane production from ruminants such as saponin, sulfate, and nitrate [3, 4]. To reduce methane production effectively, it is essential to have a good understanding of the population and distribution of ruminal methanogens. Yanagita et al. [5] reported that 2.8% to 4.0% of microorganisms in the rumen have the ability to produce methane, while species can be cultured because of strictly anaerobic characteristics. Studies relating to the 16 s rRNA gene of the archaea and methyl-coenzyme M reductase gene (*mcrA*) found that the abundant methanogenic groups in the rumen are *Methanobrevibacter*, *Methanomicrobium* and *Rumen cluster C (RCC)* [4, 6–8].

As a potential ruminal methane inhibitor, nitrate could change the rumen bacterial community composition in steers (Lin et al., 2013) in the following two ways: 1) toxicity by nitrite, an intermediate of nitrate reduction; 2) competition for hydrogen. Even though methane production is undesirable, methanogens play an irreplaceable role in maintaining rumen function by consuming hydrogen produced through rumen fermentation. Studies have suggested that nitrate- and nitrite-reducing microbes compete with methanogen for hydrogen in the rumen, making nitrate an alternative hydrogen sink that mitigates against ruminal methane production [9, 10]. The theory behind this is that nitrate reduction is energetically more beneficial than methanogenesis in thermodynamics [11]. This is because the electrochemical reduction of 1 mol nitrate to ammonia consumes 8 mol of electrons resulting in hydrogen formation [12]. Researchers believe that nitrate could greatly decrease ruminal methanogen population in unadapted ruminants [13, 14]. However, in practice, it is controversial whether nitrate can persistently inhibit methane production and methanogen populations in nitrate-adapted ruminants. Shi et al. [15] reported that the suppression effect disappeared after adaptation to nitrate, while van Zijderveld et al. found that nitrate could effectively and persistently suppress ruminal methane production in dairy cows [13]. These contrasting results could be due to the different methanogen diversities and structures in wither and dairy cattle [4]. However, limited studies have been conducted to assess the methanogenic communities in nitrate-adapted beef cattle.

This study was conducted to investigate methane production from Limousin-crossed steers fed with or without nitrate using an in vitro gas production technique. Methanogen abundance and diversity was also examined using real-time quantitative PCR and high-resolution technology Hiseq sequencing to understand the changes caused by nitrate addition and to ascertain the reasons why methane production could be inhibited by nitrate.

## Methods

### Animals

The study treatments and rations have previously been described in a report by Zhao et al. [16]. Briefly, six Limousin × Jinnan crossbreed steers (450 ± 20 kg) with permanent rumen fistula, located at Jiweifuren feedlot, were fed with mixed rations consisting of 30% concentrate and 70% corn straw. In addition, three levels of nitrate were added (on a dry matter basis): 0% (0NR), 1% (1NR), and 2% (2NR). Urea was added to 0NR and 1NR regimens to maintain the iso-nitrogen level among treatments (Table 1). Two-week adaptation was adopted for steers and ruminal microbes before sampling, and nitrate addition through 1NR and 2NR treatments was gradually increased to the target level during the adaptation period in order to avoid nitrite accumulation in the rumen. After adaptation, rumen fluid was taken before the morning feed for in vitro gas production and 6 h post-feed for DNA extraction and methanogenic community diversity measurements. For testing of methanogen community diversity, rumen fluid was frozen immediately in liquid nitrogen after sampling and then transferred to − 80°C.

**Table 1:** Chemical composition of diets

Items	Treatments		
	0NR	1NR	2NR
Metabolizable energy (MJ/kg)	7.30	7.30	7.30
Crude protein (%DM)	11.41	11.41	11.41
Ca (%DM)	0.64	0.64	0.64
P (%DM)	0.24	0.24	0.24

### In vitro gas production technique

Rumen fluid taken before the morning feed on the fifteenth day was stored in a 39 °C pre-warmed syringe thermos, then carried immediately back to the laboratory immediately for modified in vitro gas production according to the procedure of Menke et al. [17]. The detailed procedure was previously described in Zhao et al. [18] (more information in Additional file 1). Gas from 6 h, 12 h, and 24 h incubation was sampled for methane production measurement using gas chromatography (TP-2060 T, Beijing Analytical Instrument Co., Ltd, China) equipped with a TCD detector (column: TDX-01, 1 m × 3 mm × 2 mm).

### DNA extraction and methanogen quantification

Total genomic DNA from 17 samples was extracted while one 2NR sample was excluded due to improper storage. A DNA extraction toolkit was adopted for DNA extraction (Tiangen Biotech Co., Beijing, China) combined with an oscillator (Precellys 24, Bertin Technology, Montigny-le-Bretonneux, France). Rotating speed of the oscillator was 5500 rpm with two circulations and 30 s per circulation.

Total methanogen abundance was quantified with an ABI 7300 Prism real-time PCR (ABI, Foster City, CA, USA) using SYBR Green PCR RealMaster Mix (Tiangen Biotech, co., LTD, China). Methanogen 16 s rRNA sequences were amplified using primers Met630F/Met803R (Met630F-GGATTAGATACCCSGGTAGT; Met803R-RGTTGARTCCAATTAAACCGCA) [19] on the following PCR program: one cycle of 95 °C for 15 min (initial denaturation), 35 cycles of 95 °C for 30 s (denaturation), 60 °C for 30 s (annealing) and 72 °C for 30 s (elongation), followed by a final step of 72 °C for 5 min. All real-time PCR assays were performed in triplicate.

### Hiseq sequencing and data analyses

The archaeal 16S rRNA genomic sequence was amplified with the primers Arc344F/Arc519R (Arc344F-ACGGGGYGCAGCAGGCGCGA; Arc519R-GWATTACCGCGGCKGCTG) [20]. Amplicon purification was performed using a Qiagen MinElute PCR purification kit (Qiagen, Valencia, CA, USA) and the quantity of amplicon libraries was estimated with the Qubitds DNA HS assay using a Qubit 2.0 instrument (Life Technologies, USA). Subsequently, Illumina paired-end sequencing libraries were constructed from the purified PCR products using a NEB Next® Ultra™ DNA Library Prep Kit for Illumina (NEB, USA). Quality control of the amplicon libraries was performed using BioRad Experion. Finally, the libraries were sequenced on a Illumina HiSeq 2500 platform (Illumina Inc., San Diego, USA), which was used to produce paired-end sequence reads with cyclic reversible chain termination chemistry.

Sequenced paired-end reads were merged with FLASH and then grouped according to the attached barcode with QIIME software [21]. Quality control was applied using the NGS QC Toolkit [22] with the following two criteria: 1) keeping sequences with a score of 32 consecutive bases all greater than 27; 2) removing chimeras. All samples were subsampled to equal size of 300,000 reads, because an even depth of sampling is required for beta diversity calculations. The 16S rRNA operational taxonomic units (OTUs) were defined at the 3% dissimilarity threshold with uclust (v1.2.22). A total of 9736 OTUs were obtained for all samples and the average OTU quantity for each treatment was 2.071 for 0NR; 1946 for 1NR; and 1984 for 2NR. The number of sequences and OTUs is presented in Additional file 2: Table S1. The rarefaction curve (Additional file 3: Figure S1) and alpha diversity indices (ACE, Chao1, Shannon, and Simpson) were developed on the basis of OTUs. Furthermore, a phylogenetic tree for beta diversity metrics was generated with FastTree [23] based on the aligned sequences, and visualization was confirmed using principal coordinates analyses for the weighted UniFrac distances. The rarefaction curve indicated that a reasonable number of individual samples had been taken. The representative sequence of each OTU, which was the most abundant sequence, was assigned to the lowest possible taxonomic rank with RDP Classifier [24], and a reference dataset from the Greengene database was used. A Venn diagram of OTUs for different treatments was plotted. Correlation coefficients between methane production and methanogenic genus were calculated using multivariate analysis of variance in SAS and results were plotted with corrplot R software [25].

### Statistical analysis

Gas composition, methanogen abundance, methanogenic community composition, and diversity indices were analyzed with a general linear effects model using SAS 9.0 (SAS Institute, Cary, NC, USA). Furthermore, linear and quadratic tendencies were analyzed. Statistical significance was set to *P* < 0.05. Differences between treatments were assessed with Duncan’s new multiple range test.

## Results

### Methane production and methanogen abundance

**Fig. 1:**
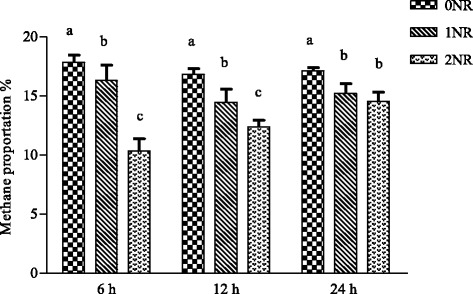
Methane proportion of in vitro gas production at 6 h, 12 h, and 24 h

**Fig. 2:**
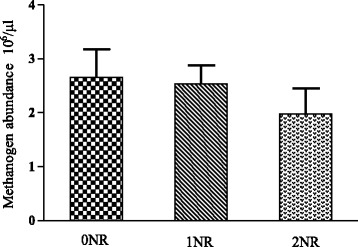
Changes in ruminal methanogen abundance by treatments

Figure 1 showed the changes in methane proportions among steers adapted to the three nitrate diets. Nitrate dramatically decreased the in vitro methane proportion of gas production at 6 h (*P* < 0.01), 12 h (P < 0.01) and 24 h (*P* = 0.01) fermentation. Ruminal methanogen population of Limousin crossed steers fed with urea was 2.66 × 106 copies/μl (Fig. 2). Nitrate linearly decreased methanogen abundance by 4.47% for 1NR and 25.82% for 2NR compared to 0NR. However, no significantly statistical difference was detected between the three treatments (*P* = 0.53).

### General information of sequencing results

**Fig. 3:**
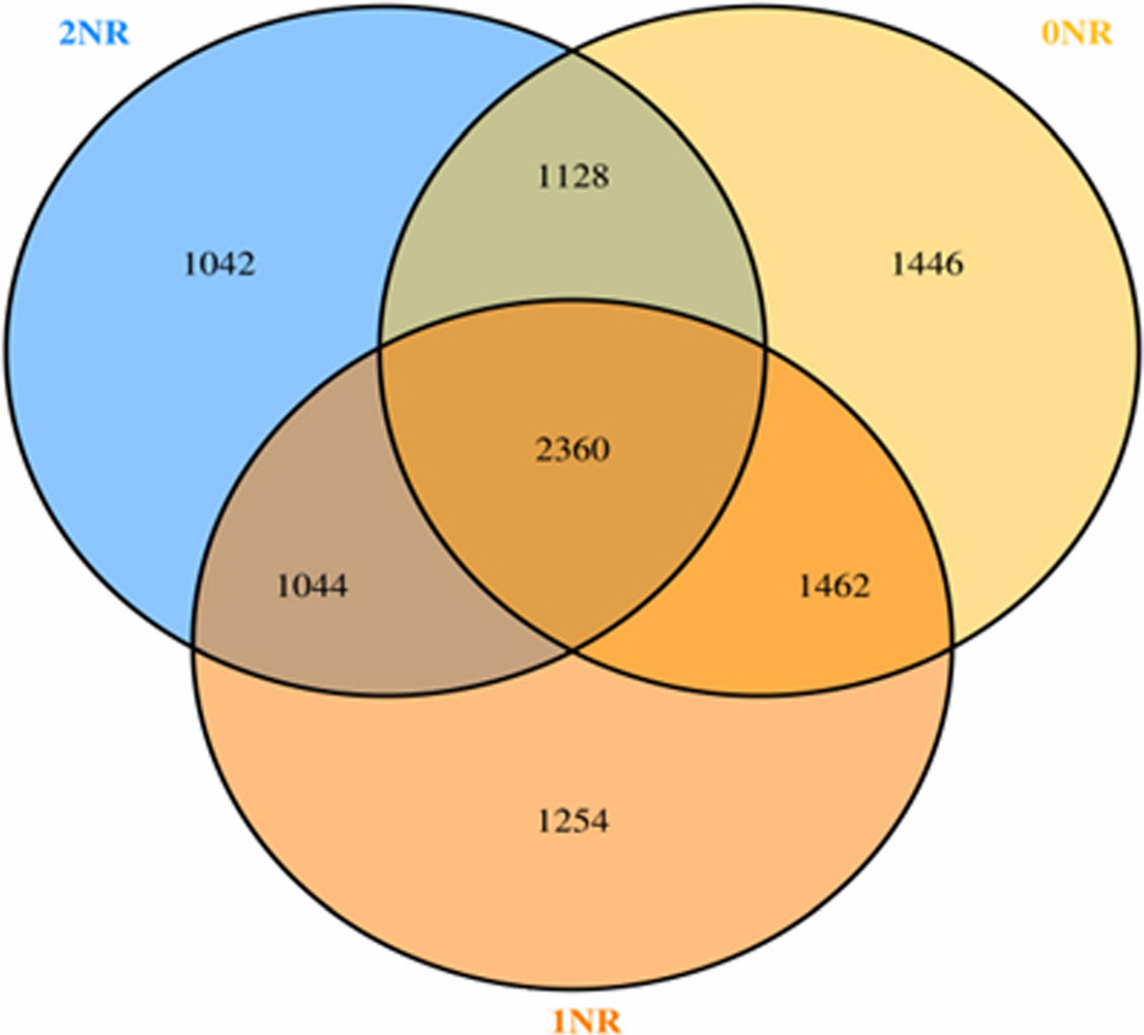
Venn diagram for overlap between observed OTUs at 3% dissimilarity of 0NR, 1NR and 2NR

In total, 9736 OTUs were obtained in this study for all samples at the 3% dissimilarity threshold. The number of OTUs exclusively found in 0NR, 1NR and 2NR treatments were 1446 (14.85%), 1254 (12.88%) and 1042 (10.70%) respectively, while shared OTUs between two treatments were 1462 (15.02%) for 0NR and 1NR, 1128 (11.59%) for 0NR and 2NR, and 1044 (10.72%) for 1NR and 2NR separately. Furthermore, there were 2360 OTUs (24.24%) shared for the three treatments (Fig. 3). The coverage of sequencing was between 99.53-99.59%, which meant that the sequencing was detailed enough to cover all 16 s rRNA sequences (Additional file 2: Table S1). The average ACE and Chao1 index of all samples was 6352 and 5789, and the average Simpson and Shannon scores were 0.86 and 3.85, respectively.

**Fig. 4:**
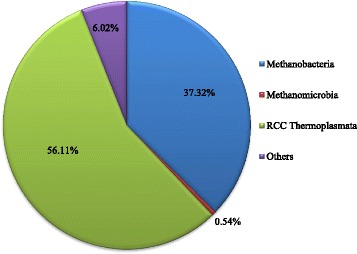
Average relative abundance of methanogenic class in the rumen of steers

Methanobacteria, Methanomicrobia, and RCC Thermoplasmata were classified in this study as the three methanogenic classes (Fig. 4). RCC Thermoplasmata and Methanobacteria were the two main classes with high abundance, accounting for 56.11% and 37.32% of total sequences, while Methanomicrobia exhibited considerably lower abundance accounting for 0.54%. In addition, approximately 6.02% sequences could not be assigned to any archaea class. Furthermore, 4 orders, 4 families and 7 genera of methanogenic groups were detected when sequences were further analyzed at a more detailed level (not shown).

At the genus level, the Methanobrevibacter from Methanobacteria and vadinCA11 from RCC Thermoplasmata were the main genera found in the samples, accounting for 35.82% and 56.09% of total sequences, respectively. In addition to the highly abundant genera, some other less prevalent genera were detected accounting for 1.25% of sequences in total. These included Methanosphaera (1.02%), Methanimicrococcus (0.22%), Methanosarcina (0.01%), Methanobacterium (0.003%), Methanoplanus (0.002%) and Methanoculleus (0.0005%).

### Effects of nitrate on methanogen composition

Statistical analysis of the relative abundance of methanogenic orders are presented in Table 2. The majority of methanogenic orders E2 and Methanobacteriales were not significantly affected by nitrate addition (*P* = 0.51; *P* = 0.42). However, Methanomicrobiales (*P* < 0.05) and Methanosarcinales (*P* = 0.01) were significantly influenced by nitrate addition, whose abundance were decreased on a linear basis and increased commensurate with increased nitrate addition (*P* < 0.05).

**Table 2:** Effects of nitrate on relative abundance of methanogen order of rumen

Items	Treatments			SEM	*P-value*	Contrast	
	0NR	1NR	2NR			*P*_*L*_	*P*_*Q*_
E2	56.15	54.15	58.34	2.62	0.51	0.55	0.32
Methanobacteriales	38.00	35.68	34.62	2.12	0.42	0.20	0.80
Methanomicrobiales	0.47^a^	0.25^b^	0.27^b^	0.06	< 0.05	0.05	0.13
Methanosarcinales	0.06^b^	0.12^b^	0.24^a^	0.03	0.01	< 0.01	0.40

*0NR* control, *1NR* 1% nitrate, *2NR* 2% nitrate, P_L_ is liner tendency, P_Q_ is quadratic tendency

Changes in methanogen genera abundance caused by nitrate are listed in Table 3. Similar to the changes in orders, the relative abundance of the majority of the genera in this study, *vadinCA11* and *Methanobrevibacter*, were not significantly influenced by nitration addition (P = 0.51; *P* = 0.49). However, the abundance of the least prevalent genera were significantly affected with increased nitrate addition. *Methanosphaera* (P_L_ = 0.0033) and *Methanimicrococcus* (P_L_ = 0.0113) abundance increased linearly commensurate with augmented nitration addition resulting in a significantly higher abundance for 2NR. With respect to low prevalence genera, growth of *Methanoplanus* was significantly suppressed by nitrate (*P* = 0.0028). The abundance of *Methanoculleus,* the smallest genus detected in this study, showed quadratic changes from 0NR to 2NR (P_Q_ = 0.0140).

**Table 3:** Effects of nitrate on relative abundance of methanogenic genera

Item	Treatments			SEM	*P-value*	Contrast	
	0NR	1NR	2NR			*P*_*L*_	*P*_*Q*_
*vadinCA11*	56.15	54.15	58.34	2.62	0.51	0.55	0.32
*Methanobrevibacter*	36.64	35.43	32.89	2.20	0.49	0.25	0.81
*Methanosphaera*	0.8100^b^	0.8550^b^	1.3575^a^	0.0977	0.0057	0.0033	0.0881
*Methanimicrococcus*	0.1000^b^	0.1017^b^	0.2300^a^	0.0333	0.0199	0.0113	0.0979
*Methanoplanus*	0.0033^a^	0.0012^b^	0.0009^b^	0.0005	0.0028	0.0013	0.1508
*Methanosarcina*	0.0029	0.0045	0.0028	0.0007	0.2141	0.9550	0.0855
*Methanobacterium*	0.0019	0.0014	0.0013	0.0004	0.4066	0.2816	0.4590
*Methanoculleus*	0.0003^b^	0.0010^a^	0.0003^b^	0.0002	0.0417	1.0000	0.0140

*0NR* control, *1NR* 1% nitrate, *2NR* 2% nitrate, P_L_ is liner tendency; P_Q_ is quadratic tendency

Based on sequencing results, nitrate did not significantly affect alpha or beta diversity of ruminal methanogen (Table 4 and Additional file 4: Figure S2). The alpha diversity indices (ACE, Chao1, Shannon, and Simpson) of the ruminal archaeal populations were not significantly affected by nitrate supplementation (*P* = 0.89; *P* = 0.66; *P* = 0.18; *P* = 0.05; Table 4). The comparisons between methanogen communities by PCoA (Additional file 4: Figure S2) based on weighted UniFrac distance revealed no difference among treatments, indicating that nitrate addition did not impact upon the ruminal archaeal population.

**Table 4:** Changes in bacterial richness and biodiversity by nitrate

Item	Treatments			SEM	*P*-value	Contrast	
	0NR	1NR	2NR			*P*_*L*_	*P*_*Q*_
ACE	6375.5	6280.1	6316.5	138.19	0.89	0.78	0.71
Chao1	5643.7	5513.9	5684.1	145.64	0.66	0.84	0.38
Shannon	0.86	0.85	0.87	0.01	0.18	0.36	0.10
Simpson	3.86	3.75	3.95	0.05	0.05	0.23	0.03

*0NR* control, *1NR* 1% nitrate, *2NR* 2% nitrate; P_L_ is liner tendency; P_Q_ is quadratic tendency

### Relationships between methane production and methanogen genera

**Fig. 5:**
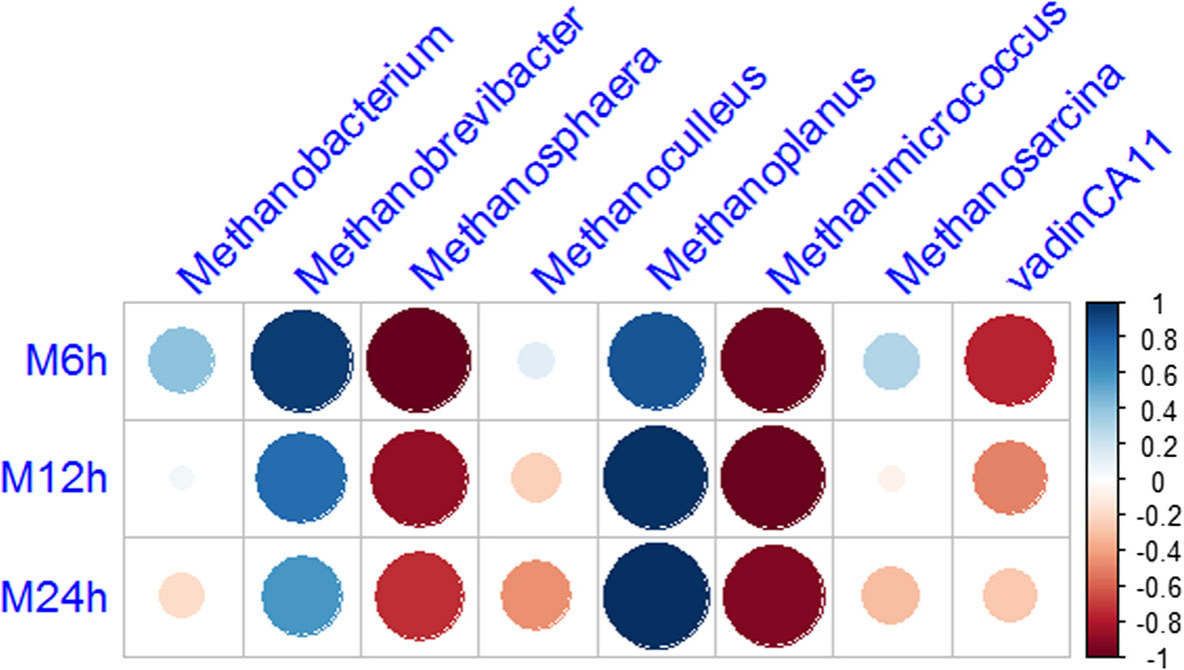
Correlation between methane production and methanogen genera abundance

Correlations between methane production and abundance of the methanogen genera were presented in Fig. 5. *Methanobrevibacter* from *Methanobacterials* and *Methanoplanus* from *Methanomicrobials* were positively related with in vitro methane production at 6 h, 12 h, and 24 h. *Methanobrevibacter* had a strongly positive relationship with methane production at 6 h and 12 h (R^2^ = 0.89; R^2^ = 0.59), while *Methanoplanus* abundance had a persistently positive correlation with methane production (R^2^ = 0.73; R^2^ = 0.97; R^2^ = 0.99). *Methanosphaera* from *Methanobacterials* (R^2^ = 0.98; R^2^ = 0.77; R^2^ = 0.54) and *Methanimicrococcus* from *Methanomicrobials* (R^2^ = 0.95; R^2^ = 0.98; R^2^ = 0.86) were negatively correlated with in vitro methane production at 6 h, 12 h, and 24 h. *VadinCA11* from the *E2* order was negatively correlated with methane production at 6 h (R^2^ = 0.59).

## Discussion

Manipulating ruminal methane production is a research focus of several disciplines between ruminant nutrition and environmental science. In the rumen, methanogenesis evolves from carbon oxide, acetic acids, and methanol by methanogens. For most methanogens, methanogensis from CO_2_ and H_2_ is the sole energy source. The process of methanogensis is affected by many environmental factors, such as carbohydrate type, digestion passage rate, and feed additives, as well as internal factors. These include: 1) the inhibition of methanogen growth and abundance, 2) the reduction of hydrogen donors and or hydrogen competition from other electron acceptors and 3) gene expression and activity inhibition of critical enzymes [3, 26, 27]. The mechanisms to decrease ruminal methane production vary but provision of hydrogen acceptors to compete with electrons as a methane precursor is an effective method [28]. Nitrate has a higher electron competing ability compared with carbon dioxide making it an effective enteric methane production inhibitor [13, 29]. Aside from the toxicity of nitrite, the intermediate of nitrate reduction in the rumen, it is also considered effective in methane inhibition. Consistent with previous results of van Zijderveld et al. [30], we found that nitrate could persistently reduce methane production of steers after a two-week adaptation period in this study. However, Shi et al. [11] observed conflicting results where inhibition disappeared when sheep were adapted to nitrate rations. These conflicting results might result from the different animal types, diet base, and environments where these studies were performed.

As previously described, methanogenesis generated from CO_2_ and H_2_ is the sole energy source for most methanogens. Therefore, consistently adding methane inhibitors to ruminants will mitigate methane production and result in inhibited growth of methanogens [4]. Nitrate can effectively suppress methane production because its reduction is a more favourable pathway for the hydrogen sink than the reduction of carbon dioxide to methane [13], resulting in decreased ruminal methanogen abundance. Furthermore, the extent of decline is more obvious with increased levels of nitrate that are added [14]. Therefore, methanogen abundance numerically decreased when nitrate was introduced to nitrate-adapted steers but not significantly, which means methane reduction is not due to total population shifts between ruminants fed with and without nitrate.

Methanogens are taxonomically categorised as *Methanobacteria*, *Methanococci*, *Methanomicrobia*, *Methanopyri* and *RCC* [32]. However, few classes have been consistently found at high abundance because of the different animal species, diets, environments, and DNA extraction methods [31–34]. More detailed assessment of rumen fluid samples using Hiseq sequencing technology provided a more intricate view of the archaea than DDGE and TGGE methods [34]. *RCC Thermoplasmata*, *Methanobacteria* and *Methanomicrobia* were the three main archaeal classes in Limousin crossed steers, accounting for 99.46% of all methanogens. At the genus level, *vadinCA11* from *Thermoplasmata* and *Methanobrevibacter* from *Methanobacteria* were the top two abundant genera in the rumen, which is different from other studies [6, 34]. Consistent with the results of Pei et al., nitrate addition did not greatly change the alpha and beta diversity of ruminal methanogens [7]. However, the taxonomic community was slightly changed by nitrate addition.

We postulate that the mechanism of nitrate influencing methanogen abundance could be hydrogen competition and nitrite toxicity to methanogens [14]. Nitrate is more competitive for hydrogen than CO_2_ and methanol [13], which could decrease energy provided for methanogen metabolism and growth. As previously mentioned, most methanogens attain their energy from CO_2_ and methanol reduction, which means that most methanogens are bacteria, which require hydrogen as a source of nutrition. These include *Methanobrevibacter*, *Methanosphaera*, *Methanoplanus*, *Methanobacterium,* and *Methanimicrococcus* [6]. Nitrate decreased the abundance of *Methanoplanus*, *Methanobacterium,* and *Methanobrevibacter* significantly or numerically due to hydrogen competition. The numerical decline of *Methanobacterium* and *Methanobrevibacter* results from high variation between duplicates within treatments. *VadinCA11* from *RCC Thermoplasmata,* the most abundant genus in this study, does not have a representative species within culture [35]. Its energy resource is methylamine but this has not been proven to be hydrogenotrophic [36]. The reason for the increases in *Methanimicrococcus* and *Methanosphaera* abundance associated with 2NR is unknown.

As previously mentioned, the changes in methanogen structure caused by nitrate might be a reason for methane inhibition [37, 38]. Correlation analysis found that methane reduction caused by nitrate addition was positively related to the reduction of *Methanobrevibacter* and *Methanoplanus* abundance and negatively correlated with an increase in the *Methanosphaera*, *Methanimicrococcus,* and *VadinCA11*. However, the abundances of *Methanobrevibacter* and *VadinCA11* were numerically changed depending on treatments so the changes may not be stable. *Methanoplanus*, *Methanosphaera,* and *Methanimicrococcus* are found at low abundance in the rumen i.e. less than 1%. Therefore, the resultant shifts in methanogen community structure in response to nitrate treatment appear to have minor effects on methane reduction. There must be other reasons accounting for methane inhibition in the rumen by nitrate. For example, the inhibition of expression of methyl coenzyme M reductase gene and the reduction of enzyme activity could be one of the reasons. Further studies need to be performed to determine these hypotheses and an accurate correlation needs to be drawn between methyl coenzyme M reductase activity and in vivo gas production in future studies.

## Conclusion

Nitrate additive greatly reduced methane production of nitrate-adapted steers. However, its influence on methanogen abundance and diversity was minor, as it only changed the abundance of less prevalent genera, such as *Methanoplanus* (negatively) and *Methanimicrococcus* and *Methanosphaera* (both positively). Correlation analysis found that there was a strong positive relationship with *Methanobrevibacter* and *Methanoplanus* abundance, and methane reduction. However, methane reduction was negatively correlated with *Methanosphaera* and *Methanimicrococcus* abundance. However, their contribution to methane reduction was not influential because of the insignificance of *Methanobrevibacter* between treatments and the low abundance of *Methanoplanus*, *Methanosphaera* and *Methanimicrococcus*. Therefore, further studies are required to consider other factors and their contribution to methane mitigation. An example could be gene expression and activity of key enzymes of methanogenesis (methyl coenzyme M reductase) in nitrate-adapted animals.

## Additional file

Additional file 1: In vitro gas production technique. (PDF 140 kb)

Additional file 2: **Table S1.** Reads and OTUs abundance of 16S rRNA gene libraries. (PDF 99 kb)

Additional file 3: **Figure S1.** Rarefaction curve for each sample with 97% similarity as threshold. (PNG 149 kb)

Additional file 4: **Figure S2.** Principal coordinates analysis showing relationships of methanogen abundance among treatments. (PNG 31 kb)

## Abbreviations

ACE: Abundance-based coverage estimator
DGGE: Denaturing gradient gel electrophoresis
DM: Dry matter
OTUs: Operational taxonomic units
PCoA: Principal coordinate analysis
PCR: Polymerase chain reaction
SEM: Standard error of mean
