# Supplementary material for: Nitrate decreases ruminal methane production with slight changes to ruminal methanogen composition of nitrate-adapted steers
Source: BMC Microbiol. 2018 Mar 20;18:21. doi: 10.1186/s12866-018-1164-1 (PMC5859718; doi:10.1186/s12866-018-1164-1)
Supplement: Supplementary file 1 — In vitro gas production technique. (PDF 140 kb) [file 12866_2018_1164_MOESM1_ESM.pdf]

In vitro gas production technique

Eighteen dried samples (200 mg DM) from 0NR, 1NR and 2NR respectively were weighed accurately into 100 mL calibrated glass syringes (HFT000025; Häberle Maschinenfabrik Gmbh, Laichingen, Germany) with a piston lubricated with Vaseline. Collected rumen fluid from 6 steers of each treatment was mixed with buffer solutions separately. Subsequently, 30 mL buffered rumen fluid was dispensed into pre-warmed syringe (39 ℃) filled with corresponding diet, and then syringes were incubated in a water bath shaker at 39 ℃ for 6 h, 12 h and 24 h. Blanks were set for each treatment and each target time using syringes with 30 mL inoculum without diet. Syringes were placed to ice water to terminate fermentation at target time for gas composition measurement. Two-millilitre gas was sampled using 5mL airtight syringe and measured for gas composition with gas chromatography (TP-2060T, Beijing Analytical Instrument Co., Ltd, China) equipped with a TCD detector (column: TDX-01, 1m × 3mm × 2mm).
