# Supplementary material for: Nitrate decreases ruminal methane production with slight changes to ruminal methanogen composition of nitrate-adapted steers
Source: BMC Microbiol. 2018 Mar 20;18:21. doi: 10.1186/s12866-018-1164-1 (PMC5859718; doi:10.1186/s12866-018-1164-1)
Supplement: Supplementary file 2 — Table S1. Reads and OTUs abundance of 16S rRNA gene libraries. (PDF 99 kb) [file 12866_2018_1164_MOESM2_ESM.pdf]

Table S1 Reads and OTUs abundance of 16S rRNA gene libraries

Samples	Sequence	OTUs	Good's_coverage
**0NR.1**	499092	3019	0.9953
**0NR.2**	442485	2795	0.9954
**0NR.3**	612932	3417	0.9953
**0NR.4**	556290	3097	0.9954
**0NR.5**	477846	2665	0.9957
**0NR.6**	505187	2641	0.9959
**1NR.1**	541544	2927	0.9956
**1NR.2**	446120	2423	0.9958
**1NR.3**	450276	2553	0.9957
**1NR.4**	410842	2323	0.9957
**1NR.5**	409021	2457	0.9955
**1NR.6**	429454	2498	0.9957
**2NR.1**	395761	2453	0.9955
**2NR.2**	477296	2649	0.9956
**2NR.3**	359668	2283	0.9956
**2NR.4**	339869	2077	0.9957
**2NR.5**	383939	2356	0.9957

0NR: control; 1NR: 1% nitrate; 2NR: 2% nitrate.
