# Supplementary figures and images for: Nitrate decreases ruminal methane production with slight changes to ruminal methanogen composition of nitrate-adapted steers
Source: BMC Microbiol. 2018 Mar 20;18:21. doi: 10.1186/s12866-018-1164-1 (PMC5859718; doi:10.1186/s12866-018-1164-1)

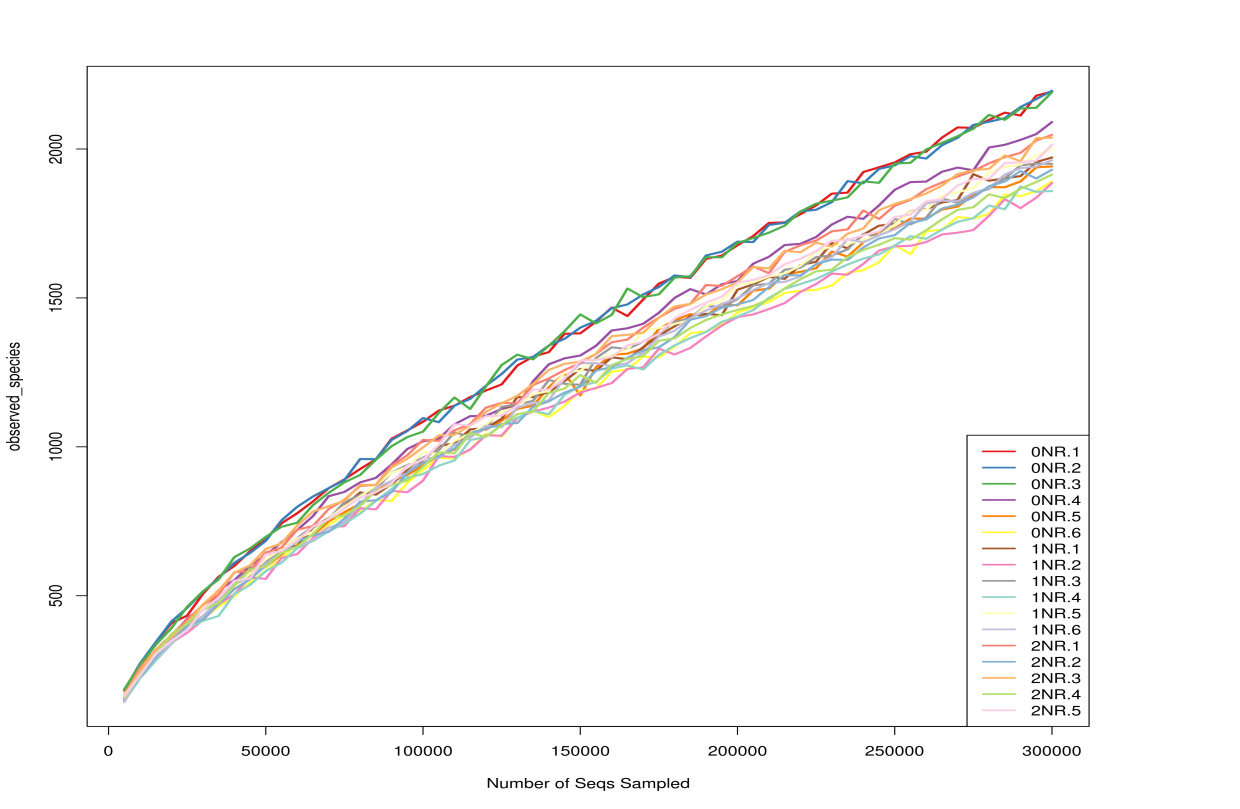

Supplement: Supplementary file 3 — Figure S1. Rarefaction curve for each sample with 97% similarity as threshold. (PNG 149 kb) [file 12866_2018_1164_MOESM3_ESM.png]

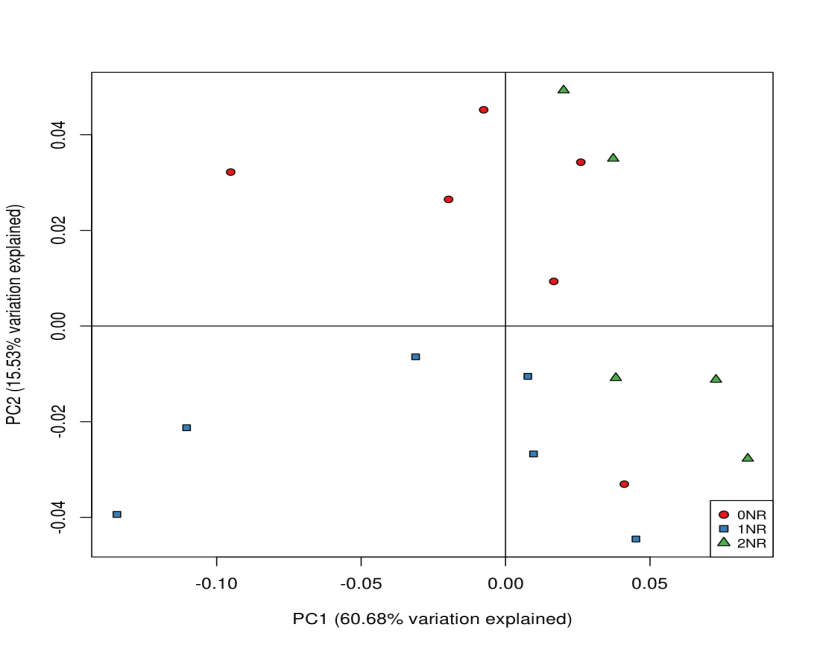

Supplement: Supplementary file 4 — Figure S2. Principal coordinates analysis showing relationships of methanogen abundance among treatments. (PNG 31 kb) [file 12866_2018_1164_MOESM4_ESM.png]
